# Shiga Toxin Type 2dact Displays Increased Binding to Globotriaosylceramide *in vitro* and Increased Lethality in Mice after Activation by Elastase 

**DOI:** 10.3390/toxins5112074

**Published:** 2013-11-08

**Authors:** Joshua C. Bunger, Angela R. Melton-Celsa, Alison D. O’Brien

**Affiliations:** Department of Microbiology and Immunology, Uniformed Services University, 4301 Jones Bridge Road, Bethesda, MD 20814, USA; E-Mails: joshua.bunger.ctr@usuhs.edu (J.C.B.); angela.melton-celsa.ctr@usuhs.edu (A.R.M.-C.)

**Keywords:** Shiga toxin 2d, Stx2dact, globotriaosylceramide/Gb3, Stx2dact binding, Stx2dact A_2_ subunit

## Abstract

Shiga toxin type 2dact (Stx2dact), an Stx2 variant originally identified from *Escherichia coli* O91:H21 strain B2F1, displays increased cytotoxicity after activation by elastase present in intestinal mucus. Activation is a result of cleavage of two amino acids from the C-terminal tail of the A_2_ subunit. In this study, we hypothesized that activation leads to increased binding of toxin to its receptor on host cells both *in vitro* and *in vivo*. To test this theory, Stx2dact was treated with elastase or buffer alone and then each toxin was assessed for binding to purified globotriaosylceramide (Gb3) in an enzyme-linked immunosorbent assay, or cells in culture by immunofluorescence, or flow cytometry. Elastase- and buffer-treated Stx2dact were also evaluated for binding to mouse kidney tissue and for relative lethality in mice. We found that activated Stx2dact had a greater capacity to bind purified Gb3, cells in culture, and mouse kidney tissue and was more toxic for mice than was non-activated Stx2dact. Thus, one possible mechanism for the augmented cytotoxicity of Stx2dact after activation is its increased capacity to bind target cells, which, in turn, may cause greater lethality of elastase-treated toxin for mice and enhanced virulence for humans of *E. coli* strains that express Stx2dact.

## 1. Introduction

Shiga toxin (Stx) is a ribosome-inactivating holotoxin with an enzymatically active A subunit and five binding B subunits (reviewed in [1,2]). The B homopentamer binds the toxin to the host cell receptor, Gb3 and, to a lesser extent, globotetraosylceramide (Gb4) [3,4,5,6]. Once toxin is bound to a target cell, it can be internalized in either a clathrin-dependent or a clathrin-independent endocytic mechanism [7]. In sensitive cells, toxin is trafficked to the Golgi, where cleavage of the A subunit occurs at the C-terminus to create a 28 kDa A_1_ subunit and a 4 kDa A_2_ subunit that remain covalently linked by a disulfide bond [8]. The A_1_ subunit contains the catalytic domain and the A_2_ subunit remains threaded through the B pentamer. Toxin continues its retrograde trafficking to the endoplasmic reticulum where the A_1_ subunit disassociates from the A_2_ and B pentamer; the A_1_ subunit then modifies the 28S rRNA of the 60S ribosome by cleavage of an adenine residue [9,10]. This process leads to permanent inhibition of protein synthesis and target cell death [11]. 

There are two major serogroups of Stx that have similar modes of action but are antigenically distinct. Stx and Stx type 1 (Stx1) are produced from *Shigella dysenteriae* type 1 and certain strains of *E. coli* (STEC), respectively; Stx type 2 (Stx2) is expressed by some isolates of STEC [12]. A single isolate of STEC may produce one or more type of Stx. In humans, infection and intoxication with STEC can cause diarrhea, hemorrhagic colitis, or, in a minority of cases, the life-threatening hemolytic uremic syndrome that may lead to acute kidney failure [13,14]. Individuals infected with STEC strains that make Stx2 are more likely to develop severe disease than those that make Stx1 only [15]. Within the Stx2 serogroup, there are many subtypes [16]; of these, the prototypic Stx2a, as well as Stx2c and Stx2dact are linked to disease in humans [17]. STEC strains that produce Stx2dact are highly virulent in streptomycin-treated mice with an oral 50% lethal dose (LD_50_) of <10 colony-forming units (CFUs). Conversely, STEC strains that make Stx2a or Stx2c (or both) are either not virulent or require about 10^10^ CFUs to reach an oral LD_50_ in streptomycin-treated mice [18].

Stx2dact was originally isolated from *E. coli* O91:H21 strain B2F1 that contains two copies of *stx*_2d_ [5,19]. Stx2dact is unique among the Stxs in its capacity to display increased cytotoxic activity for Vero cells when incubated with mouse or human intestinal mucus [20]. This increased cytotoxic activity (hereafter called activation) is due to the cleavage by elastase, contained in the mucus, of two amino acids, glycine and glutamic acid (GE), from the C-terminal end of the A_2_ subunit of Stx2dact [21,22]. 

In this study, we tested the hypothesis that removal of the C-terminal GE from the A_2_ of Stx2dact by elastase results in an increase in the capacity of the toxin to bind to target cells, and that it is this enhanced cell binding that leads to an increase in cytotoxic activity. This theory is based in part on the prediction from the Stx2a crystal structure that the C-terminus of the A_2_ subunit that threads though the B pentamer interferes with one of the B subunit receptor-binding sites [23]. Thus, we speculated that elimination of two amino acids from the C-terminal A_2_ tail might permit greater exposure of the B subunits to cell receptors. We found that elastase-activated toxin Stx2dact exhibited greater binding to both purified Gb3 as well as cells in culture when compared to Stx2dact treated with buffer. We further demonstrated that this increased binding phenotype translated to an increased lethality for mice. These results not only provide further evidence in support of a role for the A_2_ subunit in both binding and toxicity of Stx but also indicate a mechanism for the enhanced toxicity of Stx2dact after exposure to intestinal mucus.

## 2. Results

### 2.1. Binding of Stx2dact to Purified Gb3 in an Enzyme-Linked Immunosorbent Assay (ELISA)

**Figure 1:**
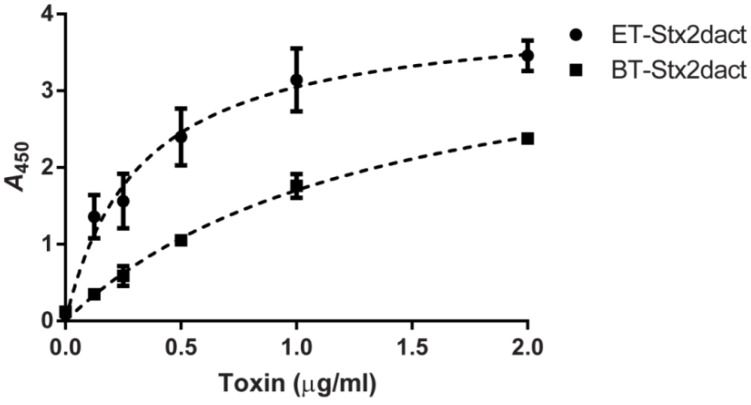
Comparative binding of elastase-treated (ET)- and buffer-treated (BT)-Stx2dact to Gb3. Different amounts of toxin were allowed to bind 1 µg of Gb3 fixed to each well of a 96-well plate. The calculated apparent *K_d_* of ET-Stx2dact was 0.32 µg/mL with a 95% CI of 0.19–4.45 µg/mL and a B_max_ of 4.0 and an R^2^ value of 0.94. The calculated apparent *K_d_* of BT-Stx2dact was 1.4 µg/mL with a 95% CI of 1.0–1.8 µg/mL and a B_max_ of 4.1 and an R^2^ value of 0.99. Mean and standard deviation are shown, and the dotted line depicts the approximate curve determined by “One site—Specific binding” in GraphPad Prism (see Experimental Section). Each value represents the mean of triplicate samples. A representative experiment is presented from three replicates.

One explanation for the enhanced cytotoxicity of Stx2dact for Vero cells that occurs after the toxin is exposed to elastase is that the activated toxin may bind with greater affinity to Gb3 than does non-treated toxin. To test this theory, we evaluated how well elastase-treated (ET) Stx2dact bound to purified Gb3 in an ELISA when compared to buffer-treated (BT) Stx2dact. Both toxins bound Gb3 in a dose-dependent manner as reflected by an increase in absorbance as toxin concentrations increased (Figure 1). When ET-Stx2dact was applied to Gb3 at the same concentration as BT-Stx2dact, the absorbance of the activated toxin was greater. Regression analyses were used to calculate the apparent *K_d_* for ET- and BT-Stx2dact bound to Gb3. For ET-Stx2dact these values were 0.32 µg/mL with a 95% confidence interval (CI) of 0.19–0.45 µg/mL and a maximum specific binding (B_max_) of 4.0 with a 95% CI of 3.5–4.6. For BT-Stx2dact these values were 1.4 µg/mL with a 95% CI of 1.0–1.8 µg/mL, and a B_max_ of 4.1 with a 95% CI of 3.5–4.7. From these results we concluded that ET-Stx2dact binds Gb3 with greater affinity than does BT-Stx2dact. Different concentrations of ET- and BT-Stx2dact were also evaluated for binding to 1 µg of purified Gb4 in an ELISA, but neither toxin bound Gb4, even at the highest concentration tested (2 µg/mL; data not shown). Together, these results indicate that activation of Stx2dact by elastase increases its capacity to bind purified Gb3 but not purified Gb4.

### 2.2. Binding Pattern of Stx2dact to Vero Cells by Immunofluorescence (IF)

**Figure 2:**
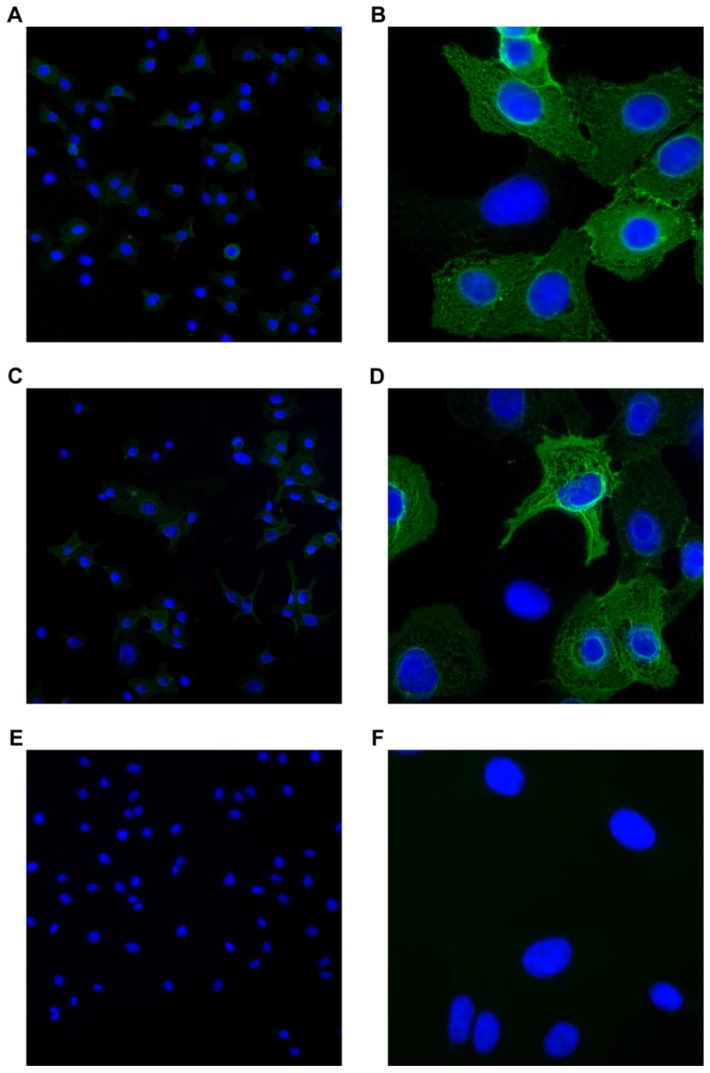
Fluorescence binding patterns of ET- and BT-Stx2dact on Vero cells. Vero cells were treated with 200 ng of ET-Stx2dact + 11E10 antibody (**A**, **B**), BT-Stx2dact + 11E10 antibody (**C**, **D**), or 11E10 antibody alone (**E**, **F**). An Alexa Fluor 488 secondary antibody was used to visualize bound toxin that had been mixed with the primary 11E10 antibody. **A**, **C**, and **E** show 100× magnification. **B**, **D**, and **F** show 400× magnification. Cells were counterstained with 4’,6-diamidino-2-phenylindole (DAPI).

Next we wanted to compare the binding patterns on Vero cells of ET- and BT-Stx2dact by IF to detect any altered or unique binding phenotypes that might occur after toxin activation. When Vero cells were incubated with ET-Stx2dact, approximately half of the cells stained positive for toxin (Figure 2A). Upon increased magnification of toxin-stained cells (Figure 2B), the fluorescent pattern detected was diffuse with a webbed-lattice appearance; no distinct membrane localization bias was noted. Similarly, approximately half of Vero cells treated with BT-Stx2dact stained positive for toxin (Figure 2C). Upon closer inspection of those cells (Figure 2D), the fluorescent pattern mimicked that of Vero cells treated with ET-Stx2dact. Vero cells treated with only the anti-Stx2a A subunit antibody 11E10 and secondary antibody showed very little background (Figure 2E,F). Thus, activation does not appear to alter the Vero cell binding pattern of Stx2dact.

### 2.3. Binding of Stx2dact to Vero Cells Measured by Flow Cytometry

**Figure 3:**
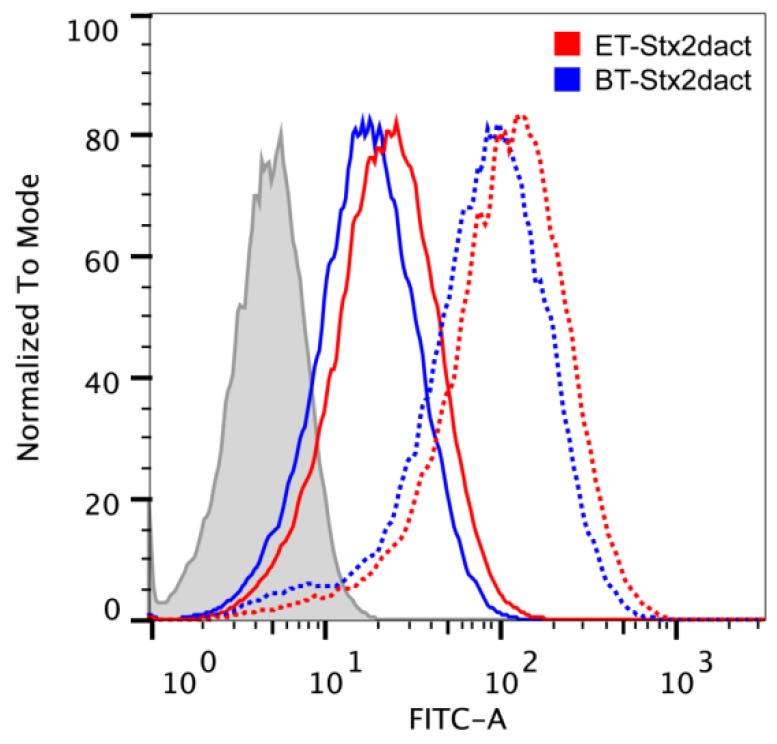
Flow cytometric profiles of ET- and BT-Stx2dact binding to Vero cells. Vero cells were treated with either no toxin (grey), 250 ng (dotted lines), or 31.3 ng (solid lines) of ET- or BT-Stx2dact. With 250 ng of toxin, we observed a right shift in both populations with a MFI of 113 and 86.5 for ET- and BT-Stx2dact, respectively. With 31.3 ng of toxin, we observed a right shift in both populations with a MFI of 22.4 and 17.4 for ET- and BT-Stx2dact, respectively.

After we observed similar binding patterns of ET- and BT-Stx2dact to the surface of Vero cells, we sought to quantitate the extent of binding of toxin to cells by flow cytometry. When Vero cells were treated with 250 ng, we observed a right shift in the total population of cells incubated with either ET- or BT-Stx2dact with a median fluorescence intensity (MFI) of 113 and 86.5, respectively (Figure 3, dotted lines). Moreover, we observed that more Stx2dact bound to each individual cell after activation. We did not observe a bimodal distribution of either population of toxin-treated cells; we interpreted these findings to mean that a majority of Vero cells bound toxin whether activated or not. We then hypothesized that intoxication with such a large dose of toxin might result in converging peaks and that there may be a maximum threshold of the amount of toxin that could be used to observe differences between ET-Stx2dact and BT-Stx2dact binding to Vero cells by flow cytometry. However, when Vero cells were exposed to a much lower dose of toxin, 31.3 ng, we continued to observe a right shift in the total population of cells incubated with either ET- or BT-Stx2dact with an MFI of 22.4 and 17.4, respectively (Figure 3, solid lines). Again, we observed that more Stx2dact bound to each individual cell after activation but did not observe a bimodal distribution in either population of toxin-treated cells. In addition, since dose-dependency was observed for toxin binding whether treated with elastase or buffer, we were unable to make statistical comparisons of percent-positive populations. Thus, we conclude from the flow cytometry data that ET-Stx2dact has a greater capacity to bind individual Vero cells than does BT-Stx2dact. 

### 2.4. Intoxication of Chemically-Treated Vero Cells with Stx2dact

**Figure 4:**
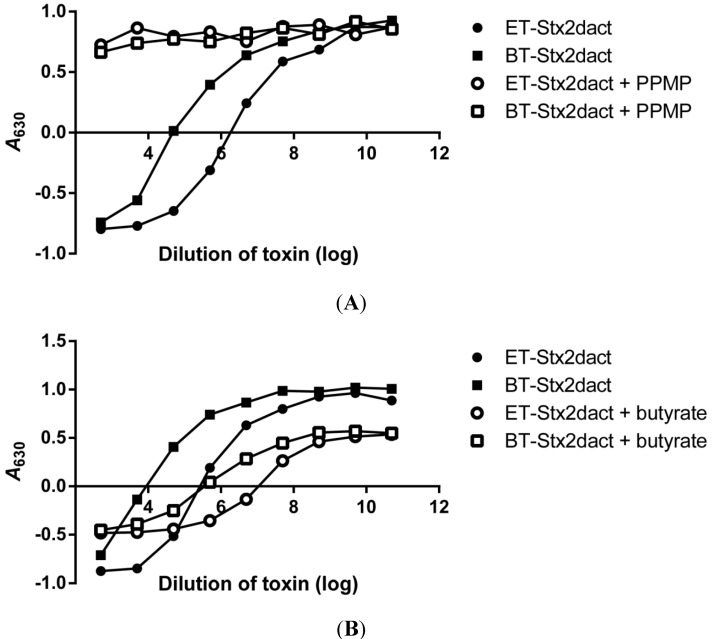
Comparison of ET- and BT-Stx2dact cytotoxicity for Vero cells treated with an inhibitor of GSLs or an enhancer of Gb3 expression. Cells were incubated with media alone or PPMP (**A**), a compound that decreases expression of GSLs, including Gb3, or butyrate (**B**), a short-chain fatty acid that increases expression of Gb3, and then either ET- or BT-Stx2dact was added. The higher the *A*_630_, the more fixed and crystal violet-stained cells remain after toxin exposure. Representative data are presented from a total of six experiments.

Next we wanted to determine if the enhanced binding capacity of ET-Stx2dact for Vero cells compared to BT-Stx2dact could be attributed directly to glycosphingolipid (GSL) expression on cells and not a different class of receptors for activated Stx2dact. d,l-threo-1-Phenyl-2-hexadecanoylamino-3-morpholino-1-propanol (PPMP) is a known inhibitor of GSL synthesis that is a structural analog of ceramide [24,25]. PPMP inhibits the glycosylation of ceramide to glycosylceramide; the latter is the precursor of GSLs of the ganglio-, lacto-, and globo-series (such as Gb3) of glycolipids. Therefore, we treated Vero cells with PPMP for 24 hours before the addition of ET- or BT-Stx2dact. In a representative experiment, Vero cells treated with PPMP were resistant to both ET- and BT-Stx2dact-mediated killing at all toxin doses tested. As expected, Vero cells not treated with PPMP were sensitive to the cytotoxic effects of both toxins (Figure 4A). The specific activities (95% CI) of ET-Stx2dact and BT-Stx2dact were 5.2 × 10^7^ CD_50_/mg (3.7 × 10^7^–7.4 × 10^7^ CD_50_/mg) and 1.7 × 10^6^ CD_50_/mg (1.1 × 10^6^–2.7 × 10^6^ CD_50_/mg), respectively, with an average activation of 33-fold over five experiments. Therefore, we concluded that binding of ET-Stx2dact to Vero cells with subsequent cell death requires expression of GSL by the cells and that activation of Stx2dact does not expand the receptor binding profile of the activated toxin compared to BT-Stx2dact. 

To further substantiate that the enhanced binding capacity of activated Stx2dact to Vero cell was directly related to the established Stx receptor Gb3, Vero cells were treated with butyrate for 24 hours and then exposed to toxin. Butyrate is a short-chain fatty acid that is known to upregulate the expression of Gb3 on the surface of cells [26,27,28]. In a representative experiment, Vero cells treated with butyrate showed increased sensitivity to cell death for both ET- and BT-Stx2dact compared to Vero cells without butyrate (Figure 4B). Vero cells treated with butyrate and then exposed to BT-Stx2dact became on average 16-fold more sensitive to toxin compared to untreated Vero cells, whereas butyrate-treated Vero cells exposed to ET-Stx2dact became on average 11-fold more sensitive toxin compared to untreated Vero cells over four experiments. These results show that Vero cell sensitivity to Stx2dact and activated Stx2dact is highly dependent on Gb3 expression. 

### 2.5. Stx2dact Cytotoxicity on HCT-8 Cells

**Figure 5:**
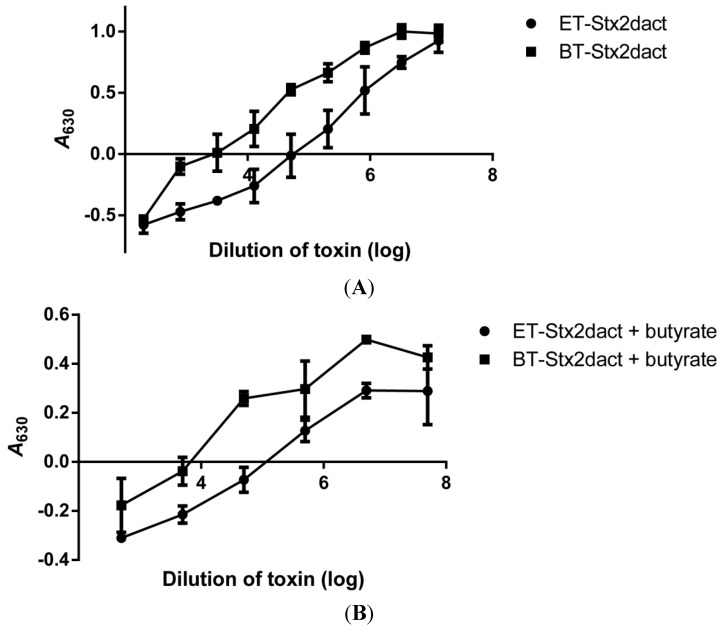
Comparison of ET- and BT-Stx2dact cytotoxicity for HCT-8 cells with or without pre-treatment by butyrate. (**A**) Toxicity assays for untreated HCT-8 or (**B**) cells pre-treated with butyrate. Mean and standard deviation are shown from duplicate samples. The higher the *A*_630_, the more fixed and crystal violet-stained cells remain after toxin exposure.

We next wanted to determine whether activation of Stx2dact could enhance its capacity to bind to human colonic epithelial cells. We chose the human colonic epithelial cell line HCT-8. This line expresses high levels of Gb4 and low levels of Gb3 [29]. In a representative experiment, we found the specific activity of ET- and BT-Stx2dact was 1.2 × 10^6^ CD_50_/mg and 5.6 × 10^4^ CD_50_/mg, respectively, with a 21-fold increase in cytotoxicity observed for ET-Stx2dact compared to BT-Stx2dact (Figure 5A). HCT-8 cells were greater than 40-fold less sensitive to intoxication as compared to Vero cells, as has been shown in other studies [29]. In addition, we found that amount of cell killing in the HCT-8 cytotoxicity assays was variable, thus, only a representative experiment is shown, and no comparative statistics were performed. Nonetheless, activation of Stx2dact was observed with an increase in cytotoxicity for HCT-8 after elastase treatment. 

Since HCT-8 cells were less sensitive to toxin compared to Vero cells, we asked whether an increase in Gb3 expression by treatment with butyrate would increase the susceptibility of those colonic cells to intoxication as we observed with Vero cells. Therefore, we treated HCT-8 cells with butyrate for 24 hours and then exposed cells to either ET- or BT-Stx2dact. In a representative experiment, we found that butyrate increased the sensitivity of HCT-8 cells to intoxication for both ET- and BT-Stx2dact (Figure 5B). The specific activity for ET- and BT-Stx2dact was 4.6 × 10^6^ CD_50_/mg and 1.3 × 10^5^ CD_50_/mg, respectively. These findings reveal that activated Stx2dact has a greater capacity to bind Gb3 not only on Vero cells, but also on human colonic epithelial cells and that increased Gb3 expression on these HCT-8 cells correlates with increased sensitivity to intoxication by Stx2dact. 

### 2.6. Neutralization of Stx2dact by Anti-Stx2a B Subunit Antibodies

**Figure 6:**
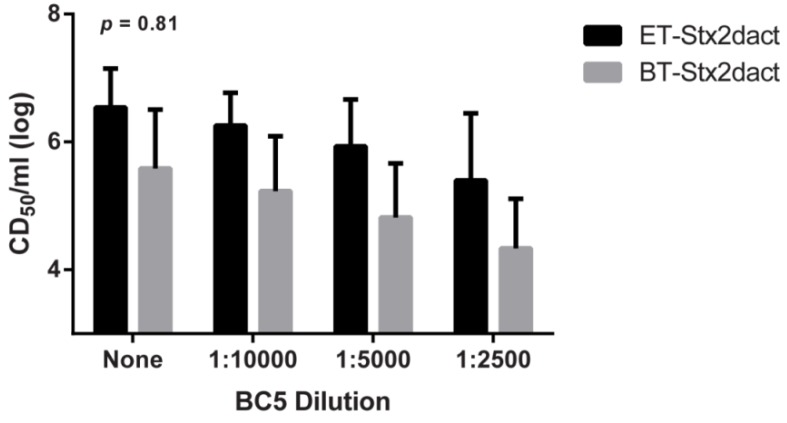
Neutralization of ET- and BT-Stx2dact cytotoxicity when incubated with different dilutions of the anti-Stx2a BC5 mAb. Two-way repeated measures ANOVA showed no statistical interaction between ET- and BT-Stx2dact for all dilutions of antibody. Experiment was done in triplicate with geometric mean and 95% CIs shown.

As previously mentioned, elastase treatment of Stx2dact results in the cleavage of two C-terminal amino acids from the A_2_ subunit. We reasoned that such changes might not only expose cryptic, or obstructed, binding domains within the B subunits but may also change the conformation of interactions between the A_2_ and B subunits. Thus, we speculated that activation of Stx2dact might render the toxin more resistant to neutralization by monoclonal antibodies that target the B subunit. We tested this idea by conducting ET- and BT-Stx2dact cytotoxicity neutralization assays with the monoclonal antibody BC5 BB12 (BC5) that targets the B subunit of Stx2a. Unpublished data from our laboratory suggest that the BC5 antibody neutralizes Stx2a by inhibiting its capacity to bind to cells as we were unable to detect toxin bound to Vero cells when probed with BC5 by fluorescence microscopy [30]. When a single dose of either ET- or BT-Stx2dact were exposed to different dilutions of BC5, a dose-dependent decrease in cytotoxicity was observed for both toxins (Figure 6). A two-way repeated measures analysis of variance (ANOVA) indicated there was no statistical difference (*p* = 0.81) in the extent of neutralization of the two toxins by BC5 antibody at all dilutions tested. These data reveal that removal of two C-terminal amino acids from the A_2_ by Stx2dact by elastase does not alter its capacity to be neutralized by the B subunit-specific antibody BC5, a finding that suggests that activation does not affect the availability of the neutralizing epitope on the toxin B subunit. 

### 2.7. LD_50_ of Stx2dact in CD-1 Mice

We next assessed whether activation of Stx2dact impacts toxicity *in vivo*. In three independent experiments, CD-1 male mice were intoxicated intravenously (IV) with various amounts of ET- or BT-Stx2dact, or with PBS alone by tail vein. By combining three independent experiments with a total of 120 mice, we determined by probit regression analyses that the LD_50_ value for ET-Stx2dact was 1.4 ng with a 95% CI of 0.91–2.1 ng, and for BT-Stx2dact was 2.5 ng with a 95% CI of 1.5–3.7 ng. Therefore, ET-Stx2dact displayed a 44% decrease in the dose required for lethality compared to BT-Stx2dact. Although the LD_50_ CIs overlapped and were therefore not statistically different, the results followed the same trend of increased toxicity and binding for ET-Stx2dact compared to BT-Stx2dact. These results further support the hypothesis that increased binding of activated Stx2dact may play a direct role in toxicity *in vitro* and as well as mortality *in vivo*. 

### 2.8. Stx2dact Binding to Mouse Kidney

**Figure 7:**
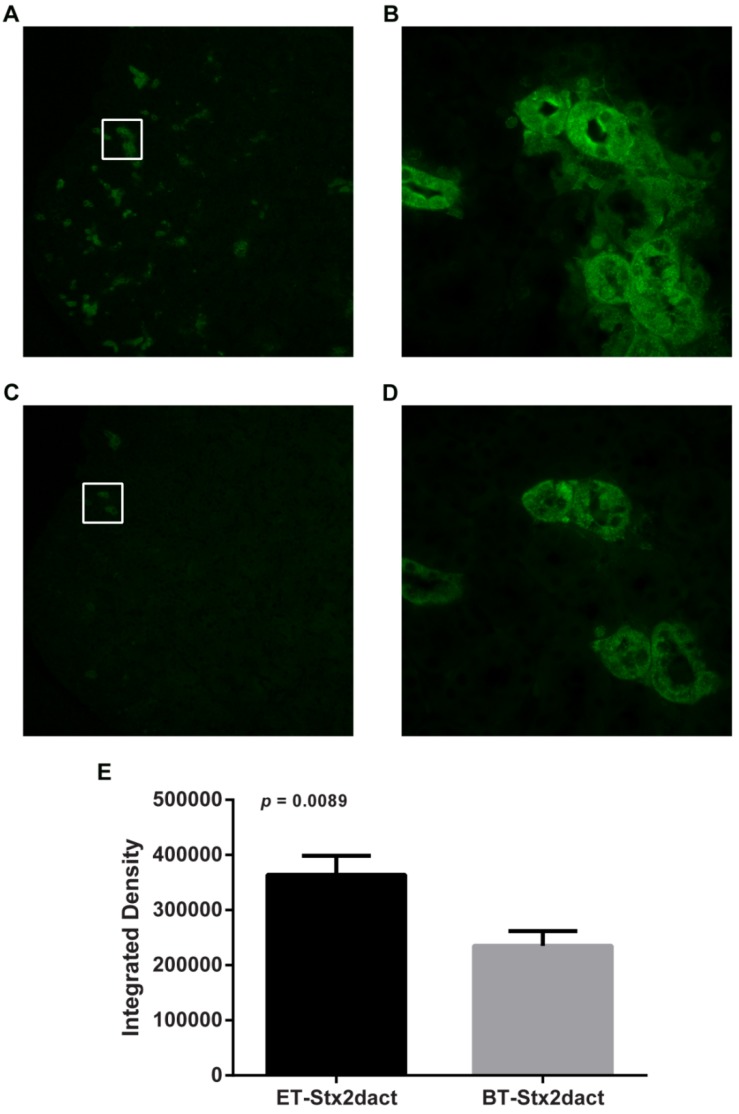
Binding of ET- or BT-Stx2dact overlaid onto fixed mouse kidney serial sections. Bound toxin was detected with rabbit-polyclonal anti-Stx2a antibody and then stained with Alexa Fluor 488-goat anti-rabbit secondary antibody. (**A**) ET-Stx2dact-exposed kidney tissue at 40× magnification and (**B**) increased magnification of white-boxed section of ET-Stx2dact-treated tissue at 400× magnification; (**C**) BT-Stx2dact-exposed section at 40× magnification and (**D**) increased magnification of white-boxed section BT-Stx2dact-treated tissue at 400× magnification. (**E**) ET-Stx2dact-stained tissue mean intensity compared to BT-Stx2dact. Mean integrated density was calculated from images **B** and **D** by selecting 10 serial regions. A Mann-Whitney U test was done and error bars show standard error of the mean.

We next postulated that the increase in ET-Stx2dact mouse lethality as compared to that of BT-Stx2dact may be reflective of an increased capacity by activated toxin to bind tubular epithelial kidney cells. To test this idea, serial sections of fixed mouse kidneys were exposed to 400 ng of ET- or BT-Stx2dact. Tissue exposed to ET-Stx2dact stained positive in numerous locations in the cortical region of the kidney but did not stain positive in the medulla (Figure 7A,B). Areas on the section that stained positive were located between the epithelial cells and the luminal space of the tubules and included those that both contained a brush border and those that did not, indicative of both distal and proximal tubules, respectively. Tissue exposed to BT-Stx2dact had a similar staining phenotype as that seen with ET-Stx2dact (Figure 7C,D). However, tissue exposed to BT-Stx2dact had fewer areas that stained positive, and areas that did so were significantly less intense with a *p* value of 0.0089 (Figure 7E). Overall, these results show that ET-Stx2dact has a greater capacity to bind tubular epithelial cells in the kidney *ex vivo* than does BT-Stx2dact. These findings also support the concept that enhanced target cell binding as a consequence of Stx2dact activation may play a role in increased lethality in mice. 

## 3. Discussion

In this study, we present results in support of one possible mechanism for the increased cytotoxicity and lethality of activated Stx2dact: enhanced binding of toxin to its receptor. While we previously showed that activation of Stx2dact leads to increased cytotoxicity for Vero cells, here we report that activation directly correlates with increased binding to purified Gb3, Vero cells, and epithelial cells in the kidney. Moreover, we found that this increased binding of Stx2dact corresponds with increased cytotoxicity for cells and increased lethality in mice. 

We initially anticipated that if increased binding to target cells was the reason for the increase in cytotoxicity of Stx2dact after activation, we would either see a different binding pattern on target cells and/or an increase in binding of ET-Stx2dact compared to BT-Stx2dact or both. None of the data we accrued supported a role for a different functional receptor after activation of Stx2dact. Indeed, both the binding phenotype by immunofluorescence and flow cytometry for cells treated with ET- and BT-Stx2dact were similar. One difference we did note between the two imaging assay methods was that on examination of cells by immunofluorescence, a subset of cells were not visibly stained for toxin whether the toxin was ET-Stx2dact or BT-Stx2dact. By contrast, no toxin-negative populations of cells were evident by flow cytometry when Vero cells were intoxicated with either ET-Stx2dact or BT-Stx2dact. Since all Vero cells showed a toxin-dependent increase in MFI by flow cytometry, we surmised that toxin was bound to all cells in each treatment group. Since all cells in each population were toxin-positive, we deemed it inappropriate to calculate a difference in the percent toxin-positive populations between groups. We speculate that these differences in positively stained cells between the two imaging methods likely reflect a difference in sensitivity of the techniques. Even with that caveat, the staining patterns were similar for the toxins when assessed by each assay method. Thus, activation of Stx2dact did not result in a different binding pattern for the toxin on cells. We therefore conclude that activated toxin binds to the same Gb3 receptor as does non-activated toxin but, from the Gb3 ELISA results and the MFI on flow cytometry for the population of cells treated with each dose of ET-Stx2dact compared to BT-Stx2dact, that binding is of higher affinity.

Although there was an increase in binding of Stx2dact toxin to Vero cells after activation, the extent of that increase did not reflect the 20-fold average enhancement in cytotoxicity. We propose two possible reasons for this quantitative difference. First, the assays are not measuring comparable activities. Toxicity assays reflect the end result of toxin binding to target cells, becoming internalized, and killing cells as a consequence of the enzymatic domain of the toxin finding and interacting with the 60S ribosome. Shiga toxin is more potent then diphtheria toxin [31] and for the latter toxin, only one molecule is needed to kill a cell [32]. Therefore, there may be hundreds of binding events to every one cytotoxic event. Therefore, if some of the Stx2dact binding events do not lead to internalization, then receptor attachment would be a less sensitive method of predicting toxicity than cell killing. Additionally, there may be a minimum dose of bound toxin molecules that directly lead to cell death; furthermore, even if toxin is bound and subsequently internalized, it may be alternatively trafficked [33,34,35] and fail to deliver the A_1_ domain of the toxin to its 60S ribosomal target. A second explanation for the relatively modest increase in activated Stx2dact cell binding, compared to the enhancement of cytotoxicity, may be that not only does the activated toxin bind better than the non-activated toxin but it may undergo alternate post-binding events compared to BT-Stx2dact that increase the likelihood of cell killing. We did not test that idea in this study. 

The reasons for the larger difference in toxicity observed for ET- Stx2dact *versus* BT- Stx2dact when tested on cells in culture *versus* lethality in mice may again reflect the nature of the assays. The cytotoxicity assay requires pg levels of toxin to kill cells whereas ng amounts are required for lethality studies. Thus Vero cell killing is a more sensitive assay than the LD_50_ analysis. However, when we overlaid ET- or BT-Stx2dact directly onto mouse kidney tissue, we did observe a clear difference in binding between the activated and non-activated toxins. Again this difference between the *in vitro* and *in vivo* assays likely reflects the difference in adding the toxin directly to the cells as compared to injection of the toxin into the mouse after which the toxin has to travel through the bloodstream, a step that may reduce the overall amount of toxin that reaches the kidneys. Moreover, there may be a minimum number of binding events that lead to intoxication and renal tubular necrosis *in vivo* for both activated and non-activated toxins. Lastly, non-activated toxin may become activated *in vivo* which would diminish differences in outcome between Stx2dact that is treated with elastase prior to injection and Stx2dact that is not so treated.

The activation of Stx2dact leads to the permanent removal of glycine and glutamic acid from the C-terminal end of the A_2_ subunit and thus an increase in cytotoxicity. The idea that the A_2_ subunit can have direct effects on binding or cytotoxicity of Stx is not unique [22,23,36,37]. Moreover, others have shown that sequence differences in the A_2_ peptide of cholera toxin and heat-labile enterotoxin can be associated with differential toxin activity. Within Stx2 subtypes, Stx2dact and Stx2c have the same B subunit sequence and only contain two amino acid differences within the A_2_ subunit that are located on the binding side of the B pentamer. These two changes alone are responsible for the large differences in cytotoxicity and lethality observed between activated Stx2dact and Stx2c [22,38]. Because activation of Stx2dact results in altered binding to Gb3, we speculated that modification of the A_2_ subunit of Stx2dact may change the overall conformation of the B subunits and ultimately the way it interacts with Gb3. However, the observation that ET-Stx2dact remains fully susceptible and indistinguishable from BT-Stx2dact to neutralization by BC5 antibody makes conformational changes as a consequence of activation a less likely scenario. One other possibility for why activation of Stx2dact leads to a greater capacity to bind could be due to the removal of a charged amino acid. However, binding events that are a result of steric effects *vs*. charge effects may be difficult to differentiate due to the dependency of cytotoxicity on the A_2_ subunit. Regardless, the removal of the C-terminal two amino acids from the Stx2dact A subunit further supports the idea that the A_2_ peptide of certain AB5 toxins can extend beyond the B pentamer and can have a distinct interaction with the capacity of the toxin to bind target cells. These findings, in aggregate, provide insight into the increased disease severity observed in humans who are infected with Stx2dact-producing *E. coli*.

## 4. Experimental Section

### 4.1. Toxin Purification

Stx2dact was isolated from *E. coli* DH5α carrying a recombinant plasmid, pSQ543, that encodes the *stx*_2d_ gene [39]. Cell-associated Stx2dact was purified by immunoaffinity chromatography with 10 mg of polyclonal rabbit anti-Stx2a [21] bound to an AminoLink Plus Immobilization column (Thermo Fisher Scientific, Rockford, IL, USA). Toxin concentration was determined by BCA assay. Toxin samples were subjected to sodium dodecyl sulfate polyacrylamide gel electrophoresis (SDS-PAGE), and the resultant gels were stained with Oriole fluorescent gel stain (Bio-Rad, Hercules, CA) to assess toxin purity. Purified toxin was stored in 1× phosphate-buffered saline (PBS) at 4 °C. 

### 4.2. Cell Culture

Vero cells (CCL-81, ATCC, Manassas, VA, USA) were maintained in Eagle’s minimal essential medium (EMEM) supplemented with 10% heat-inactivated fetal bovine serum (FBS), gentamicin (100 µg/mL), penicillin (10 U/mL), and streptomycin (10 µg/mL). HCT-8 cells (CCL-244, ATCC) were maintained in RPMI-1640 medium supplemented with 10% heat-inactivated horse serum, gentamicin (100 µg/mL), penicillin (10 U/mL), and streptomycin (10 µg/mL). Butyrate-treated cells were incubated with sodium butyrate (Sigma-Aldrich, St. Louis, MO, USA) at a final concentration of 1 mM for 24 h prior to intoxication experiments. PPMP- (Matreya, LLC, Pleasant Gap, PA, USA) treated cells were incubated at a final concentration of 8 nM for 24 h prior to intoxication experiments. Cells were grown at 37 °C in a 5% CO_2_ atmosphere. 

### 4.3. Activation Assays

Stx2dact was activated with murine elastase (Elastin Products Company, Inc., Owensville, MO, USA) in 4-(2-hydroxyethyl)piperazine-1-ethanesulfonic acid (HEPES) buffer, or treated with HEPES buffer only as a control as described previously [21]. Specifically, 200–250 ng of Stx2dact were treated with or without 5 U of murine elastase in 100 mM HEPES buffer and incubated for 1 h at 37 °C. Both samples were then treated with 200 µg of elastatinal (Sigma-Aldrich) for 1.5 h at 37 °C to stop the action of the elastase. For ELISA, IF, and flow cytometry, the monoclonal anti-Stx2a A subunit antibody 11E10 (CRL-1907, ATCC), at a final dilution of 1:100 in 1× PBS, was added during this elastatinal incubation step. The rationale for adding 11E10 at this step was to minimize the possibility that the toxin epitope recognized by that monoclonal antibody might be obscured if toxin was first allowed to bind to purified Gb3 or cells (depending on the type of assay). The level of activation of ET-Stx2dact was determined by cytotoxicity assay and compared to BT- Stx2dact. 

### 4.4. Cytotoxicity Assays

Cytotoxicity assays were done on Vero cells and HCT-8 cells. The CD_50_ was determined according to the method of Gentry and Dalrymple [40] as modified by Lindgren *et al*. [39]. Briefly, 10,000 Vero cells or HCT-8 cells were seeded into each well of a 96-well plate, and plates were incubated for 24 h at 37 °C in 5% CO_2_. Cells were then overlaid with toxin dilutions or media only and allowed to incubate for an additional 48 h. The cells were then fixed in 10% formalin, stained with 5% crystal violet in 95% ethanol, and the *A*_630_ measured as an estimate of the density of cells that remained in the wells. Values were plotted as the *A*_630_ of cells treated with a particular dilution of toxin minus half the value of the average *A*_630_ of cells treated with media only. The reciprocal of the greatest toxin dilution that caused 50% cell death was reported as the CD_50_ value. 

### 4.5. Gb3 ELISA

Purified Gb3 (Matreya) was diluted in chloroform/methanol (2:1), and 1 µg was dispensed into each well of a 96-well, U-bottom, polypropylene plate. The chloroform/methanol was allowed to evaporate overnight at room temperature. Wells were blocked with 5% BSA in 1× PBS for 1 h at 37 °C and then washed three times with 0.2 mL of 1× PBS + 0.05% Tween 20. Two-fold dilutions of either ET- or BT-Stx2dact plus 11E10 (added during the elastatinal incubation step as explained above) were added to the Gb3-coated wells to a total volume of 0.1 mL and plates were then incubated for 1 h at 37 °C. Wells were washed three times with 0.2 mL 1× PBS + 0.05% Tween 20 before the addition of 0.1 mL of goat anti-mouse-horseradish peroxidase (HRP) diluted 1:2000 in 1X× PBS (Santa Cruz Biotechnology, Inc., Dallas, TX, USA). The plates were incubated for an additional 1 h at 37 °C and wells were then washed five times in 1× PBS + 0.05% Tween 20. Next, 0.1 mL of 3,3’,5,5’-tetramethylbenzidine (TMB) peroxidase substrate solution (Bio-Rad) was added to each well, the plates were incubated for 15 min at room temperature, and the peroxidase reaction was stopped by addition of 0.1 mL 1 M H_3_PO_4_ to each well. Well contents were transferred to a 96-well clear bottom plate and the color intensity of each well at *A*_450_ was measured to quantify toxin binding to Gb3. The ELISA was done in triplicate, and the apparent *K_d_* values were calculated by application of the program “One site—Specific binding,’ which uses the same approximation as the Michaelis-Menten equation, available through GraphPad Prism 6 (GraphPad Software, Inc., La Jolla, CA, USA). 

### 4.6. Fluorescence Microscopy

Vero cells were seeded onto 4-well chambered glass slides (Nunc Lab-Tek II, Thermo Fisher Scientific) with 25,000 cells per chamber, and slides were allowed to incubate overnight at 37 °C in 5% CO_2_. The slides were then placed on ice, the cells in each chamber washed once with ice-cold 1× PBS, and then 11E10 (1:100) alone or, ET-, or BT-Stx2dact plus 11E10 in EMEM without serum were added to the cells to a total volume of 250 µL. To ensure that toxin remained on the plasma membrane of the cells, all incubations were done on ice at 4 °C. After the slides were incubated for 1 h, the cells were washed three times with ice-cold 1× PBS and fixed in 100% cold acetone for 10 min at −20 °C. Slides were removed from acetone and allowed to dry at room temperature. Next, the cells were hydrated in 1× PBS for 10 min at 4 °C and then incubated with 1% goat serum in antibody diluent reagent solution (Life Technologies, Grand Island, NY, USA) for 30 min at room temperature to block unbound sites. Cells were rinsed once with 1× PBS and stained with a 1:2000 dilution in PBS of goat anti-mouse-Alexa Fluor 488 (Life Technologies) for 1 h at room temperature. Cells were washed three times with 1× PBS, and mounted with Vectashield Hard Set Mounting Medium with DAPI (Vector Laboratories, Inc., Burlingame, CA, USA). Stained cells were viewed with a BX60 fluorescence microscope (Olympus, Center Valley, PA, USA). Images were compiled with Adobe Photoshop CS6 (Adobe Systems, San Jose, CA, USA). 

### 4.7. Flow Cytometry

Vero cells were seeded onto 6-well plates with 1 × 10^5^ cells per well then plates were incubated overnight at 37 °C in 5% CO_2_. The next day, the plates were placed on ice, the cells washed once with ice-cold 1× PBS, and then 11E10 (1:100), ET-, or BT-Stx2dact (with 11E10 added as described above) in EMEM without serum was added to the wells to a total volume of 1 mL for 1 h at 4 °C on ice. To minimize internalization of toxin all subsequent steps were performed on ice and incubated at 4 °C. Next, cells were washed three times with ice-cold 1× PBS and incubated with a 1:1000 dilution in PBS of goat anti-mouse-fluorescein isothiocyanate (FITC) antibody (BD Biosciences, San Jose, CA, USA) for 1 h. Then, cells were washed three times with ice-cold 1× PBS and treated with 0.05% trypsin-ethylenediaminetetraacetic acid (EDTA) (Life Technologies) for 10–15 min while remaining on ice. Cells were collected into 1.5 mL microcentrifuge tubes and pelleted by centrifugation for 10 min at 200 × *g*. Cell pellets were then fixed by resuspension in 2% paraformaldehyde in 1× PBS for 10 min at room temperature. Next, cells were pelleted as before, resuspended in 1 mL of 1× PBS, and passed through a 50 µm CellTrics mesh filter (Partec North America, Inc., Swedesboro, NJ, USA). Stained cells were captured with an LSR II flow cytometer (BD Biosciences) and analyzed with FlowJo v10 (Tree Star, Inc., Ashland, OR, USA). 

### 4.8. Antibody Neutralization of Activated and Non-Activated Stx2dact *in vitro*

Toxin antibody neutralization assays were done with different dilutions of the monoclonal anti-Stx2a B subunit antibody BC5 [41]. ET- and BT-Stx2dact were serially diluted into different concentrations of BC5 antibody and placed on Vero cells for 48 h as described in the cytotoxicity assay. Similarly, Vero cells were fixed, stained, and analyzed by the same procedure as the cytotoxicity assay. A two-way repeated measures ANOVA was done to compare the CD_50_ values of Vero cells with toxin and BC5 antibody *versus* the CD_50_ value of Vero cells treated with toxin without BC5 antibody. 

### 4.9. LD_50_ of Stx2dact in CD-1 Mice

Six-week-old CD-1 male mice were administered 100 µL of different dilutions of ET-Stx2dact, BT-Stx2dact, or 1× PBS + elastase + elastatinal (the latter as a control) IV through the tail vein. Three independent experiments were done to determine the LD_50_ value with a total of eight control mice and 56 mice per toxin group. Toxin doses ranged from 0.25 ng to 20 ng. Mice were observed for 12 days post intoxication and monitored for morbidity and mortality. Probit regression analyses were done with SPSS (IBM Corp., Armonk, NY, USA) to determine the LD_50_ values for each toxin group. 

### 4.10. Overlay of Stx2dact onto Kidney Tissue

Kidneys from six-week-old CD-1 male mice were collected and fixed in 10% formalin in 1× PBS and submitted to HistoServ (Germantown, MD, USA) to be dehydrated in increasing dilutions of acetone [42], embedded into paraffin, sectioned, and affixed to slides. When slides were returned, the sections were deparaffinized by submersion into Histo-Clear (National Diagnostics, Atlanta, GA, USA) two times for 10 min each. Sections were rehydrated for 20 min in 1× PBS at room temperature. Slides were then exposed to Antigen Retrieval Buffer pH 6 (Thermo Fisher Scientific) for 10 min at 95 °C. Kidney sections were then blocked with 1% goat serum in antibody diluent reagent (Life Technologies) for 30 min at room temperature. The slides were then rinsed once with 1× PBS and overlaid with 400 ng of ET- or BT-Stx2dact (without antibody) for 1 h at room temperature. Next the sections were washed three times with 1× PBS and then probed with rabbit polyclonal anti-Stx2a (1:10,000) for 1 h at room temperature. Lastly, the sections were stained with goat anti-rabbit-Alexa Fluor 488 (1:2000) (Life Technologies) and imaged with a BX60 fluorescence microscope (Olympus). Images were compiled, and integrated density was calculated with Adobe Photoshop CS6 (Adobe Systems) from 10 randomly selected positive regions of serial sections to minimized bias of selecting areas that were positive for ET-Stx2dact-treated tissue and negative for BT-Stx2dact. 

## 5. Conclusions

Overall, our findings show that increased binding of activated Stx2dact is correlated with increased cell death *in vitro* and mortality in mice. These results suggest a possible mechanism for the observed increased pathogenesis of STEC strains that produce Stx2dact in humans [43]. In conclusion, we show that the A_2_ subunit of an AB5 toxin can, not only have a significant role in Stx cytotoxicity, but also its capacity to bind to receptors. 

## Declaration

The opinions or assertions contained herein are the private ones of the authors and are not to be construed as official or reflecting the views of the Department of Defense, the Uniformed Services University of the Health Sciences, or the National Institutes of Health.
